# The Design of the Dummy Arm: A Verification Tool for Arm Exoskeleton Development

**DOI:** 10.3390/biomimetics9100579

**Published:** 2024-09-24

**Authors:** Suzanne J. Filius, Bas J. van der Burgh, Jaap Harlaar

**Affiliations:** 1 Department of BioMechanical Engineering, Delft University of Technology, 2628 CD Delft, The Netherlands; j.harlaar@tudelft.nl; 2 Department of Precision and Microsystems Engineering, Delft University of Technology, 2628 CD Delft, The Netherlands; 3 Department of Orthopedics and Sports Medicine, Erasmus MC University Medical Center, 3015 GD Rotterdam, The Netherlands

**Keywords:** dummy limb, mechanical phantom limb, exoskeleton evaluation, arm replica, upper extremity, joint impedance

## Abstract

Motorised arm supports for individuals with severe arm muscle weakness require precise compensation for arm weight and elevated passive joint impedance (e.g., joint stiffness as a result of muscle atrophy and fibrosis). Estimating these parameters in vivo, along with the arm’s centre of mass, is challenging, and human evaluations of assistance can be subjective. To address this, a dummy arm was designed to replicate the human arm’s anthropometrics, degrees of freedom, adjustable segment masses, and passive elbow joint impedance (eJimp). This study presents the design, anthropometrics, and verification of the dummy arm. It successfully mimics the human arm’s range of motion, mass, and centre of mass. The dummy arm also demonstrates the ability to replicate various eJimp torque-angle profiles. Additionally, it allows for the tuning of the segment masses, centres of mass, and eJimp to match a representative desired target population. This simple, cost-effective tool has proven valuable for the development and verification of the Duchenne ARm ORthosis (DAROR), a motorised arm support, or ‘exoskeleton’. This study includes recommendations for practical applications and provides insights into optimising design specifications based on the final design. It supplements the CAD design, enhancing the dummy arm’s application for future arm-assistive devices.

## 1. Introduction

Assistive wearable technologies, such as motorised orthosis or exoskeletons, work in parallel to the human skeleton to support or augment human motion in terms of strength, endurance, or function [1,2,3,4]. The application of such devices varies [5], from industrial [4,6] to military [7] to medical (e.g., rehabilitative [8], or daily assistive [9]) use. Developing such devices requires extensive human testing and evaluation, which brings several challenges.

The first challenge with human testing relates to safety, especially at the beginning of the development cycle, as it is difficult to predict how new sensors or software systems behave when interacting with humans. For instance, failure in safety limits or unexpected behaviour might result in fast, unexpected movements of the limb or even extend the limb beyond its natural joint limits, potentially resulting in dangerous situations. Moreover, when developing exoskeletons for a vulnerable population, such as people with severe muscle weakness or children, obtaining (medical) ethical approval and recruiting subjects that are willing to be exposed to the potential risks are time-consuming processes and are therefore not feasible for every phase of development [1]. In addition to safety issues, developing an exoskeleton is time-consuming, labour-intensive, and, therefore, costly.

Furthermore, for exoskeletons that aim to compensate for the weight of the human limb, it is essential to accurately know the properties of the limb to verify the controller’s performance. However, the properties of human limb, such as their weight and centre of mass (CoM), can only be estimated indirectly (e.g., using tables from the literature [10,11]). Especially in the case of populations with neuromuscular disorders, the anthropometrics might deviate from the available tables due to, for instance, muscle atrophy, bone density reductions, joint contractures, or deformations.

Moreover, it is difficult for humans to objectively quantify the amount of assistance provided or distinguish between small changes in controller settings, leading to subjective or even biased evaluations of the controller’s performance.

During the development of the Duchenne ARm ORthosis (DAROR), as described in [12], these challenges led to the need for a mechanical phantom. Such a phantom, like a Dummy Arm, with similar human-limb characteristics, can serve as a tool for verification for various compensation strategies, and enhances the fast iteration of exoskeleton control development [1]. The DAROR is an investigational motorised arm exoskeleton that enhances arm function. It is intended to provide assistance in daily activities for people with neuromuscular diseases such as Duchenne Muscular Dystrophy (DMD) that cope with severe muscle weakness and elevated joint stiffness, or, more accurately, ‘passive joint impedance’ (pJimp). The impedance controller of the DAROR aims to compensate for the weight of the user’s arm and the elbow pJimp (denoted as eJimp for the remainder of this work). The term pJimp describes the passive mechanical impedance against motion [13] resulting from the passive tissue around the joint and limb inertia. For instance, in people with DMD, this pJimp is elevated, presumably resulting from muscle atrophy, higher levels of connective tissue, and the development of joint contractures [14].

In the literature, few examples of dummy limbs were found, describing mannequins with a primary focus on mimicking the outer dimensions of the human limb [15,16,17]. Others, described rather complex and costly designs which equipped with sensors and actuators or soft tissues [1,5,18,19]. For the development of the DAROR, an upper-extremity phantom limb that is cost-effective, simple in design, and mimics the characteristics of the human arm, including eJimp, was required to serve as a verification tool for the compensation models.

This work presents the design and verification of a relatively simple, reproducible, cost-effective dummy arm that mimics human arm characteristics. The novelty of this design is that it allows us to replicate (elevated) eJimp and is easily adjustable in mass and CoM to fit various exoskeleton target populations. By sharing our design, we promote the development of future arm exoskeletons and facilitate the rapid iteration and objective evaluation of exoskeleton controllers.

## 2. Materials and Methods

### 2.1. Dummy Arm Design Requirements

The dummy arm was required to have the following properties:

- A representative and adjustable segment mass and CoM [10,11,20,21,22];
- A representative forearm anthropometric to fit the DAROR interface sleeve(s);
- A similar DAROR range of motion; see Table 1;
- An attachment at the base of the frame at the shoulder joint;
- An adjustable (linear) eJimp;
- An interface to attach additional objects at the wrist location.

### 2.2. Dummy Arm Design

**Figure 1:**
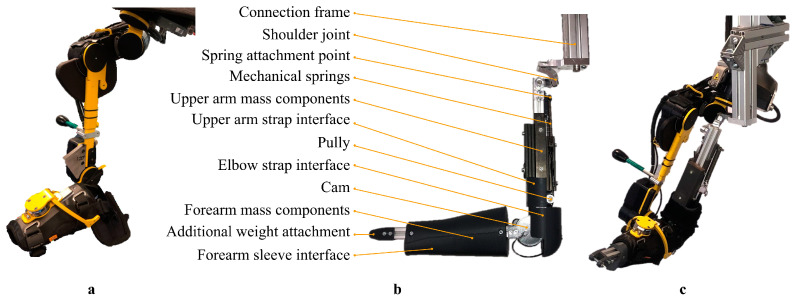
Picture of the (**a**) DAROR exoskeleton, the (**b**) dummy arm design with its components, and (**c**) the dummy arm placed inside the DAROR exoskeleton.

The design of the dummy arm can be divided into several sub-design problems: the shoulder joint, the elbow joint, the elbow joint impedance, the connection frame, and the mass of the upper and lower arm. The design of the dummy arm, with its components together with the DAROR, is shown in Figure 1.

#### 2.2.1. Shoulder Joint

The shoulder joint is a complex joint due to its high number of degrees of freedom (e.g., glenohumeral elevation and horizontal and axial rotation, according to the ISB recommendations [23,24,25]), as well as its large range of motion. A serial chain of three revolute joints was used to mimic the three degrees of freedom of the shoulder. However, with three revolute joints, several configurations are possible. Each has its advantages and disadvantages with respect to its range of motion and the possible occurrence of gimbal locking. As the range of motion is primarily restricted by the exoskeleton, which consists of three actuators mounted in series at an inclination of approximately 60° with respect to each other [12], a similar configuration for the dummy arm is used. Each of the three revolute joints consists of a set of two ball bearings (MR95-2Z) to minimise friction and provide support to possible bending moments. The joints are connected through 3D-printed or milled aluminium brackets (for the final design milled aluminium brackets were used).

#### 2.2.2. Elbow Joint

The elbow joint can be considered a hinge joint. Therefore, the design of the elbow joint is relatively straightforward, consisting of two ball bearings (625ZZ) mounted to the upper arm frame on either side of an axis and attached to the forearm frame.

#### 2.2.3. Passive Elbow Joint Impedance

**Figure 2:**
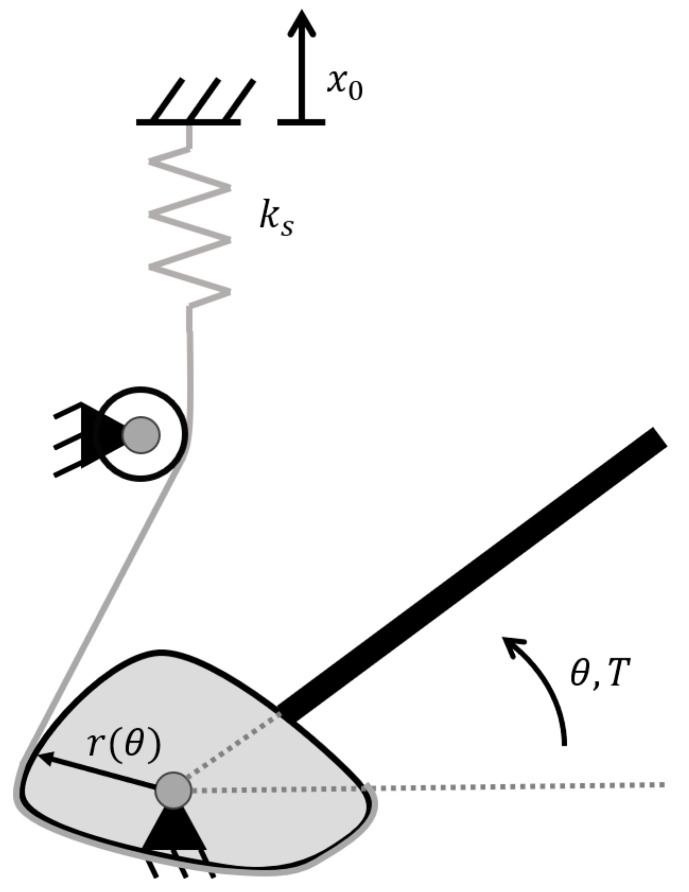
Schematic of the wrapping cam mechanism to generate the desired joint stiffness. Here, $k_{s}$ is the spring stiffness, θ the angle of the elbow, *r* is a function of θ, defining the cam shape (for the current prototype, r is constant, resulting in a circular cam), and $x_{0}$ is the pretension applied to the spring. The torque *T* can be expressed as $T=T(\theta ,k_{s},x_{0}$). A detailed discussion on deriving the cam shape for a given torque profile can be found in [27,28].

The impedance of the dummy arm elbow joint is created using a wrapping cam mechanism. This mechanism consists of a spring attached to a belt, which wraps around a cam attached to the forearm. Upon rotation of the forearm, the belt wraps/unwraps around the cam, stretching/relaxing the spring. This is shown schematically in Figure 2. A cam is attached on either side of the elbow with the belt either wrapping the cam in a clockwise or counterclockwise direction to simulate impedance during both the flexion and extension of the elbow. The current design aimed to mimic the linearised torque profile of the passive eJimp similar to [26], which is achieved using a circular cam. However, different torque profiles can be generated depending on the shape of the cam. The torque-angle profile can be further tuned by changing the spring stiffness ($k_{s}$) or the spring’s pretension ($x_{0}$). An additional advantage of the wrapping cam mechanism is that the springs can be mounted parallel to the upper arm frame, resulting in a compact design.

#### 2.2.4. Connection Frame

To connect the different components (joints, springs, and weights), a skeleton for the upper and lower arm is made of aluminium extrusion beams (20 × 20 mm), which allows the various components to be attached freely along the beam’s length. As a result, the system is modular, allowing for easy adjustment of different parts. Additionally, the dummy arm can mimic different human arm sizes using various lengths of beams. A rigid 3D-printed PLA shell is attached to the frame, which mimics the forearm [29] for interfacing with the exoskeleton sleeve.

#### 2.2.5. Mass and Centre of Mass of the Upper and Lower Arm

Steel plates are mounted to the aluminium beams to mimic the overall mass and the CoM of the human forearm and upper arm. The location for the mounting of the steel plates is based on a CAD model, which is used to estimate the CoM. The dimensions of the dummy arm are based on the biometric data from [10], resulting in an approximate length of 250 mm for the lower arm and 300 mm for the upper arm. The segment mass parameters are based on the work of [10,11,20,22], with a mass of approximately 1.6 to 2 kg for the upper arm and 0.9–1.1 kg for the forearm. The mass of the hand (ca. 400 g) is not included, but it can be attached separately alongside additional weights to mimic lifted objects in the hand if deemed necessary. The advantage of using a modular system consisting of aluminium extrusion profiles is that properties such as its mass and CoM can be easily adjusted to the desired application by removing or adding weights and shifting their position. As such, the dummy arm can be adjusted to simulate different arms.

#### 2.2.6. Verification of Design

The verification of the dummy arm consists of two parts. First, the direct measurement of the segment properties of the dummy arm is performed by weighing the segment mass and estimating the CoM in the direction of the limb with respect to the proximal joint. This latter property was measured by hanging the upper and forearm individually to a rod with two ropes connected to the endpoints of the limb segments. The location on the rod where it needed to be held fixed to keep the rod in balance (level) was taken as the CoM location. The second part verifies the realised eJimp with four different spring-type configurations. This is done using the DAROR set-up. The DAROR moves the dummy arm elbow joint (position-controlled) through a range-of-motion cycle (either ca. 10–109° or 40–116°) with approximately 8 static intervals, while the shoulder joint remained in a neutral position (ca. GH elevation: 10°, horizontal 10°, axial 0°). A video showing this procedure is available in the Supplementary Materials. The joint torque is measured by the deflection of the series elastic element in the actuators (torque accuracy 0.5 Nm, torque precision 0.4 Nm, ref. [12]) in the elbow actuator. The range-of-motion cycle is repeated with and without springs attached to the dummy arm elbow joint. First, these torque-angle profiles are fitted to a 5th-order polynomial (polyfit, polyval) using MATLAB (version 2021b). Then, these torque-angle profiles measured with and without the attached springs were subtracted to receive the measured eJimp of the dummy arm. Additionally, a first-order fit of this eJimp was created to compare the realised Jimp slope, offset, and equilibrium (zero-crossing) to the average human eJimp measured in twelve non-disabled male individuals in the study of Filius et al. [26].

## 3. Results

The dummy arm was developed and fabricated based on the design requirements. Table 2 summarizes the realised characteristics of the dummy arm in terms of its mass, CoM, and eJimp characteristics, and compares it to the intended human model and the CAD design parameters. The table shows that the mass and CoM of the realised dummy arm is comparable to the human arm model, but is tunable by adjusting the position and number of steel plates, as mentioned in Section 2.2.5.

### 3.1. Joint Impedance Realisation

**Figure 3:**
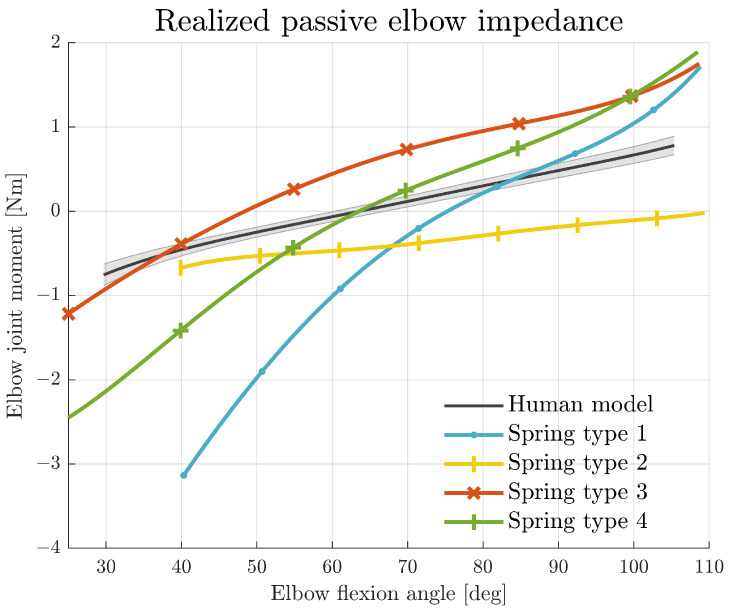
The realised passive eJimp using different spring types compared to the average human eJimp model of 12 non-disabled male individuals, with data retrieved from [26]. The solid grey line represents the group average, and the shaded area shows the standard error of the mean.

The realised eJimp in the dummy arm is compared to the measured human eJimp from our previous work [26] and presented in Figure 3. This previous work used an active elbow support set-up to identify the human eJimp in twelve non-disabled male individuals. The actuator placed at the elbow joint rotated the participant’s forearm over the elbow’s range of motion in the horizontal plane. In this plane, the gravitational component of the passive forces exerted on the elbow joint remains constant. A six-degrees-of-freedom force/torque sensor on the forearm interface sleeve measured the elbow joint moments, representing the human eJimp [26]. Figure 3 shows the human eJimp model as a group average and standard error of the mean of the identified human eJimp and compares this to the realised eJimp of the four spring types.

Depending on the selected spring type and pre-tension of the springs, different eJimp characteristics were realised; see Table 3. Spring type 4 has the most representative equilibrium point (i.e., where the torque-angle curve crosses the zero-line) compared to the human model. The slope of type 2 is more representative of the slope of the non-disabled eJimp, whereas the slope of spring type 4 could represent an elevated eJimp.

### 3.2. Activities of Daily Living

The achieved range of motion of the shoulder joints is larger than the shoulder range of motion of the DAROR set-up, and the elbow joint is similar to the range of motion of a human arm in the DAROR set-up [12]. This achieved range of motion is sufficient to reach the desired activities that are considered relevant for daily living for this application. To illustrate the sufficient range of motion of the dummy arm in the DAROR set-up, some static poses of the relevant activities of daily living are displayed in Table 4. Moreover, the dummy arm design allows for the attachment of an additional object at the wrist location, which is illustrated in the third column (c), where a 200 g mass is attached to the attachment point to simulate a lifted object in hand.

### 3.3. Cost-Analysis

The material costs for the various components of the dummy arm are listed in Table 5. Here, labour costs are not included in this overview, as they are dependent on the available facilities. Moreover, the costs of a 3D-printed shoulder joint are considered. However, a milled aluminium shoulder joint will be more expensive, especially due to the involved labour and equipment costs. The approximate fabrication time is around 5 h in the case of a 3D-printed shoulder joint, and for milled aluminium joints, an additional 5–10 h should be taken into account, depending on proficiency and the available equipment.

## 4. Discussion

This work aims to present the design of a relatively simple, reproducible, and cost-effective dummy arm that can be used as a verification and development tool for (motorised) upper-extremity exoskeletons. The dummy arm mimics the human arm mechanics with adjustable mass, CoM, and eJimp characteristics. The forearm design simulates the shape of a human forearm and allows for the attachment of additional weights at the wrist to simulate (objects lifted in) the hand.

### 4.1. Limitations

Some simplifications are made when comparing the dummy arm with a human arm. First of all, it is assumed that the joints are ‘ideal’ joints (e.g., having a fixed centre of rotation), whereas, in reality, human joints have complex surface geometries and show shifts in their joint axis of rotation during motion [30]. This is especially apparent for the shoulder joint, as the elevation or suppression of the scapula occurs in most elevation movements. Joint misalignment might exert excessive interaction forces on the dummy arm, especially as the dummy arm joints give no slack. Therefore, there is little compensation possible against misalignment. This has practical implications, as the 3D-printed parts at the elbow joint may fatigue or fail when subjected to substantial joint misalignment. However, as opposed to human joints, the dummy arm joint centres are more easily visually aligned with the joints of the DAROR set-up.

Since the dummy arm is not instrumented with sensors like that in the work of [5,19], no feedback on the possible effects of joint misalignment between the dummy arm and exoskeleton could be obtained. Including sensors in the design would allow us to investigate influences of joint misalignment. However, adding sensors adds complexity and costs to the design of the dummy arm. Therefore, depending on the research interest, it must be considered whether it is worth including these components, or whether a simpler version might suffice. Moreover, since human joints are not ‘ideal’ like the simplified joints of the dummy arm, the measured interaction forces in a phantom limb might deviate from real-world scenarios with a human limb.

The generalisability of the dummy arm design to other upper-extremity exoskeletons has not yet been investigated. However, by providing our CAD design in the Supplementary Materials, we enable others to make the necessary adjustments for various exoskeleton applications.

### 4.2. Recommendations

Although beyond the scope of the current study, it is of interest to investigate whether the realised eJimp characteristics of the dummy arm spring types are representative of people with neuromuscular disorders who cope with elevated eJimp. Different spring characteristics could approximate the human eJimp more closely. This requires more investigation. Potentially, the torque-angle profile of people with DMD is less likely to behave linearly. As mentioned above, the dummy arm eJimp profile can be further tuned by adjusting the shape of a cam (wrapping cam mechanism). Examples of this can be found in the work of [28], who used a serial wrapping cam mechanism to design an upper-limb assistive device. A more general description of the design and use of a wrapping cam mechanism can be found in the work of [27].

Although not of interest within the current application, several directions exist in which to improve the dummy arm. For instance, the force transmission between the dummy arm and the exoskeleton could be considered. In reality, due to the soft-tissue deformation of the skin and subcutaneous tissue, the transmission of the forces to the arm is different than when a rigid structure is used. A possible solution would be to use a soft outer layer for the dummy arm, similar to what is done by the authors of [1]. Moreover, since humans have no ideal joints, it might be worth investigating, depending on the research interest, making the joints more human-like to allow for small shifts in the joint axes of rotation. However, adding soft tissue, or more human-like joints, adds complexity and costs to the design of the dummy arm.

To reduce the costs of the design, the shoulder brackets could also be 3D-printed. Nevertheless, care should be taken with the load demands on the material in combination with the material selection and manufacturing techniques. Otherwise, a redesign of the brackets to strengthen these structures is recommended. After our initial PLA 3D-printed shoulder bracket failed, we decided to mill the shoulder from aluminium.

The advantage of using a modular dummy arm is that it can be adjusted to the anthropometrics for different populations, such as children or people with pathologies affecting the upper extremity (e.g., DMD, spinal muscular atrophy, amyotrophic lateral sclerosis, stroke). By making use of a more patient-like dummy arm, the burden on patients for the development of exoskeletons can be decreased, as the tests can be very exhaustive and demanding for patients. Testing time with vulnerable patients is also limited, while a dummy arm is always available. Therefore, using a patient-like dummy arm allows for faster assistive or rehabilitative exoskeleton development. However, the translation of the exoskeleton performance with the dummy arm to the intended target population should be further investigated.

Lastly, it is challenging to objectively compare the performance of exoskeletons with respect to each other, especially as the performance metric (e.g., perceived level of provided support) can become very subjective and differ across the reachable workspace. Using standardised dummy arm and objective evaluation methods allows for a more objective comparison of exoskeleton performance, which is relevant for the patients as well for the developers, as it can point to possible areas of improvement.

## 5. Conclusions

The current dummy arm successfully mimicked the characteristics of the human arm and was proven to be a helpful development and verification tool for the developed investigational DAROR exoskeleton. Since the design is relatively simple, reproducible, and cost-effective, it promises to serve more arm exoskeleton applications, and is therefore shared. The design allows for easy adjustment of the mechanical properties (e.g., mass, CoM), outer dimensions, and eJimp torque-angle profiles to fit the specific characteristics of a (vulnerable) intended target population. Using such dummy limbs, like the dummy arm, enhances the fast iteration and objective evaluation of new control strategies, and can save time, labour, and the burden of voluntary participants, who may be exposed to safety risks in an early stage of exoskeleton development.

## Figures and Tables

**Table 1:** DAROR shoulder range of motion.

Joint Rotation	Target
GH elevation	11° to 137°
GH horizontal	−46° to 138°
GH axial	−113° to 65°
El flexion/extension	2° to 120°

Abbreviation: GH, glenohumeral joint; El, elbow.

**Table 2:** Comparison of the mass and centre of mass (CoM) based on CAD with real-life measurements.

	Human Model		Dummy Arm		CAD	
	Mass ^a^ kg	CoM ^b^ mm	Mass kg	CoM mm	Mass kg	CoM ^c^ mm
UA	1.6–2	137	1.8	140	1.7	$\left[\begin{array}{c} 2.5\\ -140.2\\ -2.0 \end{array}\right]$
FA	0.9–1.1	106	1.0	110	1.0	$\left[\begin{array}{c} -0.2\\ -112.7\\ 2.6 \end{array}\right]$

Abbreviations: UA: Upper arm; FA: Forearm; CoM, centre of mass; eJimp, passive elbow joint impedance; z, zero-crossing (equilibrium point); M, mean; SD, standard deviation. All CoM values are expressed with respect to the proximal joint centre. ^a^ Retrieved from [10,11]. ^b^ Based on average locations of COM as ratio of segment length of dummy arm, retrieved from Table 4 of [10]. ^c^ The second element (y-axis) is aligned with the limb in a proximal direction, and the first element (x-axis) points laterally.

**Table 3:** Passive eJimp characteristics of different spring types compared to human model.

	1st-Order Fit Nm/rad *x* + Nm	z (M ± SD) deg
Human model	1.14x−1.29	66 ± 11
Spring type 1	4.31x−2.46	74
Spring type 2	1.29x+1.45	-
Spring type 3	3.04x−0.03	48
Spring type 4	3.67x−1.24	63

Abbreviations: z, zero-crossing (equilibrium point); M, mean; SD, standard deviation.

**Table 4:** Illustration of the range of motion of the dummy arm for a set of activities of daily living.

a. Tabletop Activities	b. Feeding Activities	c. Lifted Object (200 g)
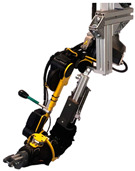	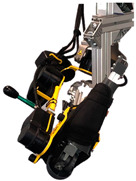	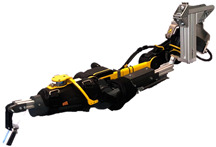
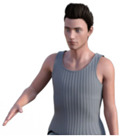	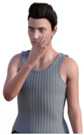	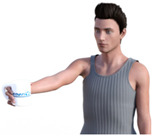

**Table 5:** Material costs of the various components. If the costs depend on the material properties, the total weight or length is reported. Dimensions of the different sub-components can be retrieved from the CAD files in Supplementary Materials S1. Costs are rounded to the nearest EUR 0.50.

Material	Costs
Bearings	EUR 13.50
Frame	EUR 6.00 (550 mm)
Mounting materials	EUR 15.00
Axes	EUR 1.00 (200 mm)
Weights	EUR 12.50 (1.9 kg)
Springs	EUR 11.00
Belt and pulleys	EUR 13.00
PLA filament	EUR 6.50 (280 g)
Total	EUR 78.50

## Data Availability

The original contributions presented in this study are included in the article and Supplementary Material, further inquiries can be directed to the corresponding author/s.

## Supplementary Materials

The CAD design of the dummy arm can be downloaded at https://doi.org/10.4121/3c4fa57d-3fc6-4e27-933c-00a02a6e5a33. A supporting video is available at https://www.mdpi.com/article/10.3390/biomimetics9100579/s1.

## Abbreviations

The following abbreviations are used in this manuscript:

pJimp	passive joint impedance
eJimp	passive elbow joint impedance
CoM	centre of mass
DAROR	Duchenne ARm Orthosis
GH	glenohumeral
CAD	Computer Aided Design

## References

[1] Barrutia W.S., Bratt J., Ferris D.P. (2023). A Human Lower Limb Mechanical Phantom for the Testing of Knee Exoskeletons. IEEE Trans. Neural Syst. Rehabil. Eng..

[2] Gull M.A., Bai S., Bak T. (2020). A review on design of upper limb exoskeletons. Robotics.

[3] Dollar A.M., Herr H. (2008). Lower extremity exoskeletons and active orthoses: Challenges and state-of-the-art. IEEE Trans. Robot..

[4] De Looze M.P., Bosch T., Krause F., Stadler K.S., O’Sullivan L.W. (2016). Exoskeletons for industrial application and their potential effects on physical work load. Ergonomics.

[5] Dežman M., Massardi S., Pinto-Fernandez D., Grosu V., Rodriguez-Guerrero C., Babič J., Torricelli D. (2023). A mechatronic leg replica to benchmark human–exoskeleton physical interactions. Bioinspiration Biomim..

[6] Koopman A.S., Kingma I., Faber G.S., de Looze M.P., van Dieën J.H. (2019). Effects of a passive exoskeleton on the mechanical loading of the low back in static holding tasks. J. Biomech..

[7] Van Dijk W., Van de Wijdeven T., Holscher M., Barents R., Könemann R., Krause F., Koerhuis C.L. Exobuddy-A non-anthropomorphic quasi-passive exoskeleton for load carrying assistance. Proceedings of the 2018 7th IEEE International Conference on Biomedical Robotics and Biomechatronics (Biorob).

[8] Plaza A., Hernandez M., Puyuelo G., Garces E., Garcia E. (2023). Lower-Limb Medical and Rehabilitation Exoskeletons: A Review of the Current Designs. IEEE Rev. Biomed. Eng..

[9] Gandolla M., Antonietti A., Longatelli V., Pedrocchi A. (2020). The Effectiveness of Wearable Upper Limb Assistive Devices in Degenerative Neuromuscular Diseases: A Systematic Review and Meta-Analysis. Front. Bioeng. Biotechnol..

[10] Chandler R.F., Clauser C.E.C., McConville J.T.J.J.T., Reynolds H.M., Young J.W., Yough J. (1975). Investigation of Inertial Properties of the Human Body.

[11] Clauser C.E., McConville J.T., Young J.W. (1969). Weight, Volume, and Center of Mass of Segments of the Human Body.

[12] Filius S.J., Keemink A.Q.L., Meijneke C., Gregor W., Kuenen T., Janssen M., Harlaar J., Kooij H.V.D. A 4DOF Impedance-Based Exoskeleton with Weight and Elbow Stiffness Compensation: For Duchenne Brooke Scale 4.

[13] Maggioni S., Melendez-Calderon A., Van Asseldonk E., Klamroth-Marganska V., Lünenburger L., Riener R., Van Der Kooij H. (2016). Robot-aided assessment of lower extremity functions: A review. J. Neuroeng. Rehabil..

[14] Filius S.J., Harlaar J., Alberts L., Houwen-van Opstal S., van der Kooij H., Janssen M.M. (2024). Design requirements of upper extremity supports for daily use in Duchenne muscular dystrophy with severe muscle weakness. J. Rehabil. Assist. Technol. Eng..

[15] Noda T., Teramae T., Ugurlu B., Morimoto J. Development of an upper limb exoskeleton powered via pneumatic electric hybrid actuators with bowden cable. Proceedings of the 2014 IEEE/RSJ International Conference on Intelligent Robots and Systems.

[16] Madani T., Daachi B., Djouani K. (2016). Non-singular terminal sliding mode controller: Application to an actuated exoskeleton. Mechatronics.

[17] Dávila-Vilchis J.M., LAZ-Avilés, Ávila Vilchis J.C., Vilchis-González A.H. (2019). Design Methodology for Soft Wearable Devices—The MOSAR Case. Appl. Sci..

[18] Toth A., Pilissy T., Bauer M.O., Al-Absi G., David S., Fazekas G. Testing the Limit Range of Motion Safety Function of Upper Limb Rehabilitation Robots with an Anthropometrically Adjustable and Sensorized Dummy Limb. Proceedings of the 2022 International Conference on Rehabilitation Robotics (ICORR).

[19] Bessler-Etten J., Schaake L., Prange-Lasonder G.B., Buurke J.H. (2022). Assessing effects of exoskeleton misalignment on knee joint load during swing using an instrumented leg simulator. J. Neuroeng. Rehabil..

[20] Contini R. Body Segment Parameters, Part II; Technical Report. https://citeseerx.ist.psu.edu/document?repid=rep1&type=pdf&doi=eb1971ff961ba567256c52fa89ad2be5e25ec9f9#page=5.

[21] Dempster W.T., Gaughran G.R.L. (1967). Properties of body segments based on size and weight. Am. J. Anat..

[22] Drillis R., Contini R., Bluestein M. (1964). Body Segment Parameters A survey of Measurement Techniques.

[23] Wu G., Van Der Helm F.C., Veeger H.E., Makhsous M., Van Roy P., Anglin C., Nagels J., Karduna A.R., McQuade K., Wang X. (2005). ISB recommendation on definitions of joint coordinate systems of various joints for the reporting of human joint motion—Part II: Shoulder, elbow, wrist and hand. J. Biomech..

[24] Doorenbosch C.A., Harlaar J., Veeger D.H. (2003). The globe system: An unambiguous description of shoulder positions in daily life movements. J. Rehabil. Res. Dev..

[25] Stienen A.H., Keemink A.Q. Visualization of shoulder range of motion for clinical diagnostics and device development. Proceedings of the 2015 IEEE International Conference on Rehabilitation Robotics (ICORR).

[26] Filius S., Janssen M., van der Kooij H., Harlaar J. Comparison of Lower Arm Weight and Passive Elbow Joint Impedance Compensation Strategies in Non-Disabled Participants. Proceedings of the 2023 International Conference on Rehabilitation Robotics (ICORR).

[27] Tidwell P.H. (1995). Wrapping Cam Mechanisms. Ph.D. Thesis.

[28] Schroeder J.S., Perry J.C., Beyerlein S.W. (2016). Application of Wrapping Cams in an Unpowered, Upper-Limb Assistive Device. Ph.D. Thesis.

[29] Story A. (2020). Right Hand Reference. https://grabcad.com/library/right-hand-reference-1.

[30] Schiele A., Van Der Helm F.C. (2006). Kinematic design to improve ergonomics in human machine interaction. IEEE Trans. Neural Syst. Rehabil. Eng..

